# Interprofessional collaboration regarding patients’ care plans in primary care: a focus group study into influential factors

**DOI:** 10.1186/s12875-016-0456-5

**Published:** 2016-05-28

**Authors:** Jerôme Jean Jacques van Dongen, Stephanie Anna Lenzen, Marloes Amantia van Bokhoven, Ramon Daniëls, Trudy van der Weijden, Anna Beurskens

**Affiliations:** Research Centre for Autonomy and Participation for People with Chronic Illnesses, Zuyd University of Applied Sciences, Nieuw Eyckholt 300, 6419 DJ Heerlen, The Netherlands; Department of Family Medicine, CAPHRI School for Public health and Primary Care, Maastricht University, Maastricht, The Netherlands

**Keywords:** Interprofessional collaboration, Multidisciplinary teamwork, Interprofessional Relations, Cooperative behaviour, Health care team, Primary health care, Qualitative research, Focus groups

## Abstract

**Background:**

The number of people with multiple chronic conditions demanding primary care services is increasing. To deal with the complex health care demands of these people, professionals from different disciplines collaborate. This study aims to explore influential factors regarding interprofessional collaboration related to care plan development in primary care.

**Methods:**

A qualitative study, including four semi-structured focus group interviews (*n* = 4). In total, a heterogeneous group of experts (*n* = 16) and health care professionals (*n* = 15) participated. Participants discussed viewpoints, barriers, and facilitators regarding interprofessional collaboration related to care plan development. The data were analysed by means of inductive content analysis.

**Results:**

The findings show a variety of factors influencing the interprofessional collaboration in developing a care plan. Factors can be divided into 5 key categories: (1) patient-related factors: active role, self-management, goals and wishes, membership of the team; (2) professional-related factors: individual competences, domain thinking, motivation; (3) interpersonal factors: language differences, knowing each other, trust and respect, and motivation; (4) organisational factors: structure, composition, time, shared vision, leadership and administrative support; and (5) external factors: education, culture, hierarchy, domain thinking, law and regulations, finance, technology and ICT.

**Conclusions:**

Improving interprofessional collaboration regarding care plan development calls for an integral approach including patient- and professional related factors, interpersonal, organisational, and external factors. Further, the leader of the team seems to play a key role in watching the patient perspective, organising and coordinating interprofessional collaborations, and guiding the team through developments. The results of this study can be used as input for developing tools and interventions targeted at executing and improving interprofessional collaboration related to care plan development.

**Electronic supplementary material:**

The online version of this article (doi:10.1186/s12875-016-0456-5) contains supplementary material, which is available to authorized users.

## Background

The number of people with multiple, chronic diseases is increasing [1, 2]. These chronic diseases lead to considerable functional, social, and emotional impairment and an increased health care demand, especially in primary care [3–5]. In addition, moving patient care out of the hospitals into the primary care setting also influences this increase. Further, it can be assumed that the needs of patients with complex health care demands often are beyond the expertise of any single profession [6, 7].

To deal with the complex health care demands and to deliver efficient, safe, and high quality care, different health care professionals need to collaborate [8–10]. This process of interprofessional collaboration (IPC) is defined by the World Health Organization as: “Multiple health workers from different professional backgrounds work together with patients, families, caregivers, and communities to deliver the highest quality of care” [8]. Instead of a fragmented health care supply of single health care professionals, IPC aims to attain more tailored and synchronized health care delivery from a diversity of disciplines [9, 11]. Within this process, the patient’s perspective plays a central role. In patient-centred care, the individual patient’s goals are at the centre of care [12].

Shared talking about (patient) goals, formulating action plans, and developing a patient-centred care plan can be considered useful strategies to integrate the patient perspective in the decision-making process. Ideally, within shared goal setting, the patient discusses and sets health-related goals together with a health care professional [13, 14]. After setting goals with the patient, the health care professional discusses these goals in the interprofessional team meeting. The team subsequently flows into action planning and negotiation on who best carries out each action [13]. Based on the patient’s goals and formulated actions, the team develops a patient-centred care plan, which can be seen as a collaborative and dynamic document [15].

In the care for chronically ill, however, collaborative goal setting and action planning have not been implemented on a large scale, and there seems to be a lack of evidence on how to integrate the patient’s perspective [15, 16]. Furthermore, the situation of a variety of single health care professionals working for their own practice makes IPC a challenge [7]. In addition, different interrelating factors can serve as barriers to or facilitators of the process of IPC. Several factors that influence IPC in primary care have been mentioned in the literature [11, 17–19]. San Martin-Rodriguez and colleagues (2005) divide these factors into issues related to interpersonal relationships (interactional determinants), conditions within the organisation (organisational determinants), and the organisation’s environment (systemic determinants) [18]. Dinh (2012) makes an approximately equal categorisation and distinguishes barriers into three levels: individual, practice, and system level [17]. From a review by Xyrichis and Lowton (2008) two main themes emerge: team structure and team processes. Team structure includes the team premises, size, and organisational support. Team processes include the team meetings, clear goals, and objectives [11]. In the literature all evidence concentrates on factors related to IPC in general. However, in the context of team talk about (patient) goals, formulating action plans, and developing a patient-centred care plan, IPC might be influenced by other dynamics and factors.

Mapping the viewpoints, barriers, and facilitators of IPC concerning patients’ goals and action plans seems to be a prerequisite for developing interventions to improve shared goal setting and action planning by IPC in primary care. Therefore, the aim of this qualitative study is to explore influential factors of IPC regarding patient goals and the patient-centred care plan.

## Methods

### Study design

We conducted a qualitative focus group study. Four focus group meetings were organized in March 2013 with experts and health care professionals from different disciplines in primary care (*n* = 31). We chose to conduct focus groups because we assumed that the interaction between the different participants could lead to more in-depth insights [20, 21]. Relevant aspects of this study are reported following the Consolidated Criteria for Reporting Qualitative Research (COREQ) [22].

### Setting

The focus group meetings took place in a quiet meeting room at Zuyd University of Applied Sciences (Heerlen, the Netherlands) and lasted approximately 90 min each.

### Participants

Participants were selected by means of purposive sampling to achieve a diverse range of health care professionals and experts from different disciplines. We aimed to include a variety in expertise in the following areas: interprofessional collaboration, self-management support, shared decision making, communication, and interprofessional education. We invited primary health care professionals from disciplines representing family physicians, practice nurses, occupational therapists, physical therapists, psychologists, and social workers. We only included health care professionals working within interprofessional teams and dealing with chronically ill patients in primary care. In sampling the participants we assured that they were appointed at a diversity of practices. We conducted two types of focus groups: groups with experts (*n* = 2) and groups with health care professionals (*n* = 2). Recruitment took place in the Netherlands, and potential participants were selected from a list either composed by the research team or named by key persons. During recruitment, we purposefully invited 27 health care professionals and 22 experts. Candidates who were interested in participating received written background information without disclosure of the exact purpose of the focus groups in order to avoid bias. Eventually, 16 experts and 15 health care professionals participated (Table 1). As presented in Table 1, 11 men and 20 women participated; the mean age was 47. Participants signed a written informed consent form and filled out an additional questionnaire with socio-demographic variables. The informed consent form was used to confirm the participants’ voluntary participation and the right to end participation in the study at any moment, if desired. Additional file 1 provides a detailed overview of the participants.

**Table 1:** Characteristics of the participants

Source	Code	N	Mean age	Men	Women
Focus Group 1 (Experts)	E1	8	40	2	6
Focus Group 2 (Experts)	E2	8	54	2	6
Focus Group 3 (Health care professionals)	H1	9	44	4	5
Focus group 4 (Health care professionals)	H2	6	52	3	3
	Total	31	48	11	20

### Data collection

A semi-structured interview guide with open-ended questions was prepared for all focus group meetings (see Additional file 2) [20]. The interview guide started with an open question to capture participants’ first thoughts about IPC. Subsequent questions were related to experiences with IPC in developing patient-centred care plans, to factors that influence the process of interprofessional collaboration, and to experienced barriers and facilitators. Before actual use, the interview guide was tested in a pilot interview and adapted where needed. An experienced and independent researcher acted as moderator, guiding the interviews and starting each focus group with a short introduction. Subsequently, he asked participants about experiences, barriers, and facilitators to the process of interprofessional collaboration on patient-centred care plan development in the primary care setting. Follow-up questions were used to gain more in-depth information. Two focus groups started discussions from the patient perspective and ended with the team perspective, and two focus groups used the opposite order. Besides the moderator, a second researcher (JvD) facilitated the meeting and took field notes.

### Analysis

To analyse the data, we applied an inductive content analysis approach [23]. The focus group meetings were audio-taped and transcribed verbatim. NVivo 9 software was used to structure the transcripts and code the data [24]. The analysis was carried out by two researchers (JvD) and (SL), both experienced in qualitative research. JvD and SL independently analysed all transcripts and carried out open coding of all quotes relevant to the aim of the study. Different concepts were identified and grouped into subcategories, (axial coding) [25]. In the next step, the two researchers compared and discussed their codes until they reached consensus and subsequently categorized the different subcategories. In case of disagreement, the research team was asked for advice. In the last step, the researchers identified different key categories into which the subcategories could be divided.

### Trustworthiness

The researchers’ field notes and written comments were used in the analysis process to enhance the trustworthiness of the study. Furthermore, two researchers coded data independently and then discussed and compared categories and subcategories. An independent senior researcher with experience in conducting and guiding focus group interviews moderated the interviews to reduce the researchers’ influence. To increase accuracy, validity, and credibility, a member check was done. Main findings were sent to all participants, giving them the opportunity to comment on the key findings. To enhance the results’ transferability, purposive sampling was used to include the perspectives of various disciplines.

## Results

**Fig. 1:**
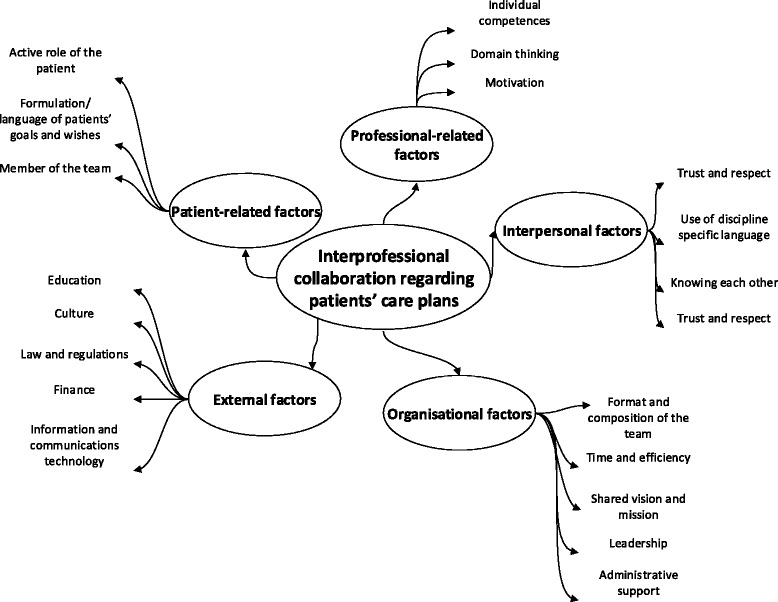
Key categories of factors

Content analysis of the four focus group meetings revealed 5 key categories of factors: (1) patient-related factors, (2) professional-related factors, (3) interpersonal factors, (4) organisational factors, and (5) external factors (Fig. 1). The categorisation of determinants of IPC as presented by San Martin-Rodriguez (2005) appeared to be useful in structuring the key findings of this study [18]. In addition to this categorisation, two categories of factors were added, related to both patients’ and professionals’ perspectives.

### Patient-related factors

#### Active role of the patient

Many participants described the importance of a central and proactive role of the patient. Patients, for example, could participate in team meetings. Prior to this, one of the participants used the term *active citizenship* in which the role of an assertive and responsible patient is highlighted. To support patient involvement and stimulate this active citizenship, one participant mentioned self-management support. According to the participants, an increasing number of courses seem available for training patients to get more in control of their own lives and stimulate communication with health care professionals.

*“The patient has to change and become more proactive as a member of the team”. (Researcher, E1.2)*

#### Formulation/language of patients’ goals and wishes

Further, a difference between patients’ and professionals’ goals was distinguished, in which participants mentioned professionals’ tendency to set goals solely from the professional perspective. Some participants stated that the wishes, needs, and problems of the patient should be translated into patient-directed goals, and they highlighted the importance of a goal-setting process. According to the participants, these goals need to be explored together with the patient before the interprofessional team meeting. Participants also stated that goals should be formulated in the patient’s experience language. After this, the patient’s goals can be introduced during the interprofessional team meeting.

#### Member of the team

Further, participants mentioned that, occasionally, the patient or a relative or informal caregiver are invited to attend interprofessional team meetings. They mentioned both benefits and barriers regarding the presence of a patient during a team meeting. Some participants experienced a participating patient as a benefit because in this way the patient’s goals can be synchronized with the goals of the different health care professionals. Furthermore, by participating, patients get the feeling of being taken seriously as members of the team, and health care professionals no longer talk *about* the patient, but *with* the patient. Other patients experience barriers, e.g., the overwhelming professional perspective of the health care providers in comparison with the patients’ perspective. As a response, one of the participants introduced *safe climate* as a factor that influences the input of the patient during team meetings.

### Professional-related factors

#### Individual competences

Participants perceived professionals’ individual competences as a factor influencing the process of collaboration. The ability of professionals to discover patients’ goals and introduce these during team meetings was discussed during one of the focus group sessions as such a competence of professionals.

*“In dialogue with the patient, professionals should discover the patients’ goals, wishes and expectations as good as possible and bring these to the interprofessional team meeting”. (Occupational therapist, H1.7)*

Other competences were related to open communication and the ability to collaborate with colleagues from other disciplines as facilitators of interprofessional collaboration.

#### Domain thinking

A professional-related barrier was *domain thinking,* or professionals only focusing on their own domains and showing a lack of interest for aspects outside these domains. One of the participants mentioned that professionals need to look beyond their own profession to share goals with colleagues from other disciplines.

*“Dare to cross the own borders”. (Occupational therapist, H1.7)*

#### Motivation

Another critical factor for successful collaboration and sustaining success for the future is related to the professionals’ continuing motivation. Participants mentioned both intrinsic and extrinsic aspects of motivation. Intrinsic motivation was described as the professionals’ personal ideological drive, need, and willingness to collaborate. Financial incentives were mentioned as an aspect of extrinsic motivation.

*“In starting up something new, everyone is motivated and willing to invest time and resources, but after a while, this willingness and motivation decrease”. (Physical therapist, H1.5)*

### Interpersonal factors

#### Use of discipline specific language

The majority of the participants mentioned one or more factors related to the interaction between team members. According to the participants, professionals from different disciplines compile an interprofessional team with a diversity of perspectives and discipline specific language. These differences in languages are expressed by some of the participants as possible barriers to collaboration and a cause for confusion.

*“Be able to define a shared language (also for the patient), and create a successful base for developing a shared care plan”. (Consultant, E1.1)*

Participants stated the importance of defining a shared language. One of the participants mentioned the World Health Organization’s International Classification of Functioning (ICF), a model used to classify patients’ functioning with standardized terminology, as a possible tool to support defining patients’ functioning from various perspectives using a mutual language [26]. In addition, participants recommended approaching patients from a broader perspective and formulating goals per discipline or domain. In addition to adopting a shared language, participants also stressed the importance of a shared interpretation of care, for instance, the concept of patient-centred care.

#### Knowing each other

According to the participants there is a difference between recently started teams and more self-regulating teams working together for a longer period. Participants were consistent about the importance of professionals (especially in beginning teams) paying attention to knowing each other as persons and knowing each other’s professional backgrounds. Participants believed that professionals knowing each other well are better able to take advantage of each other’s discipline-specific competences.

*“Collaboration contributes to the mutual respect and supports in knowing each other’s core business, strengths, and points in which professionals can reinforce each other, in the patients’ benefits”. (Social worker, H1.6)*

Also for recently started teams, participants stressed the importance of in-person meetings to learn to know each other, create an atmosphere of mutual trust, and learn about the others’ added value.

#### Trust and respect

In addition to the foregoing, trust and respect are two related terms that were mentioned multiple times during the focus groups as underlying preconditions of successful collaboration. A relation of trust and respect can grow by creating an open and safe environment in which the professionals dare to think and act broader than their own discipline. Within a safe environment, reflection and intervision were considered by participants as useful tools to talk about both individual and team functioning, roles and pitfalls, and improving the IPC. One of the participants stated the importance of periodic reflection by emphasizing that teams should regularly look at their status quo and possible pitfalls. As an example, one of the participants mentioned a technique called *mirroring,* a form of self-reflection that can be used as a method of intervision to look at both individual and team functioning.

*“Mirroring, how do I perform in comparison with my colleagues, or how does our team function, compared with other teams”? (Manager, E1.7)*

### Organisational factors

This category contains factors related to the organisation and execution of IPC regarding care plan development. Organisation-related factors were introduced by most of the participants and were associated with the format and composition of the team, time and efficiency, shared vision and mission, leadership, and administrative support.

#### Format and composition

Participants discussed a large variety of formats of a team meeting. Team meetings differed in composition, group size (3–14 members), frequency, duration, objective and mission, location and setting, and the number of patients discussed. Despite the fact that participants prefer smaller teams because of efficiency, they also stated the importance of the presence of all health care professionals involved. It seems to be hard to define who should be part of the core team. In practice, some professionals are present only on demand, based on the specific expertise needed at that moment. Further, some professionals do not take the oath of secrecy (e.g., welfare workers from municipalities), which has been mentioned as a barrier to sharing patient-related information and participation in a team meeting. Occasionally, the patient also is a member of the team.

#### Time and efficiency

Lack of time, both travelling time and the time that the health care professionals need to invest in participating in a team meeting, was considered to be a serious barrier. Due to different and busy schedules among team members, it sometimes is hard to find an appropriate meeting date.

*“I think it’s a mission impossible to assemble six health care professionals from different disciplines, meeting at the same time on a structural basis”. (Researcher, E1.3)*

Participants preferred the maintenance of short communication lines between colleagues from different disciplines. Different methods of communication concerning complex patients, both formal and informal, were introduced, e.g., emailing, phone calls, in person meetings, and virtual meetings. In addition, participants mentioned that by collaborating through virtual meetings and asynchronous communication, the time issue could be tackled.

#### Shared vision and mission

Further, participants stated the relevance of general agreement on the team’s goals and objectives, vision and mission, and how to talk as a team about patients’ goals. Participants thought that clear rules and collaboration agreements support the progress and transparency within team meetings.

#### Leadership

All agreed that one of the team members should take the role of a leader or coordinator who monitors the shared goals and objectives, and guarantees the organisational requirements. This role, comprising the planning, agenda setting, structuring, and chairing of the team meetings, is a crucial task in attaining efficient and successful team meetings, as perceived by the participants. They saw the practice nurse as an appropriate person for this leading role.

*“When the organisational task, containing the preparation and facilitation, is not arranged, the progress of the team meeting will be a disaster. In addition, the follow up of agreements needs to be agreed on”. (Occupational therapist, E1.7)*

Besides the role of a leader who carries out organisational tasks, participants also mentioned the role of a case manager being the contact person for the patient and overseeing all agreements and actions. Per patient, the team members need to consider who the appropriate case manager would be. According to the participants, this case manager explores goals and needs with the patient, and brings the patient’s goals and wishes as a patient advocate to the interprofessional team meeting.

*“Good to have a contact person for both patient and caregivers, someone who knows about the patient’s situation”. (Researcher, E1.3)*

#### Administrative support

In addition, administrative support for documenting the meetings and adjusting the patient file, was acknowledged by the participants as a supporting factor for the practical organisation of the team meeting.

### External factors

#### Education

Participants explained the importance of paying attention to interprofessional education and suggested collaboratively discussing patient cases among students from various disciplines. At present professional education is seen as discipline specific, as a result of which individual expertise varies.

#### Culture

In addition to education, cultural aspects were highlighted during the focus group meetings. The cultural aspects include, for example, the traditional dominant role of the general practitioner (GP) as a leader. Several participants experienced the traditional role of the GP as a barrier to the unrestrained contribution of team members from other disciplines.

#### Law and regulations

Participants experienced the presence of laws, rules, and regulations as a barrier to the process of collaboration. The law regarding client privacy was mentioned multiple times. Strictly, the patient must give permission for the sharing of (patient) information between health care providers. In practice, this seems unworkable and is experienced by the participants as bureaucracy.

#### Finance

Another barrier introduced by several participants was financial remuneration. For most of the professionals, except for the GP and the practice nurse, participation in an interprofessional team meeting is not being rewarded financially. Participants argue that financial compensation for all team members would motivate and facilitate collaboration. As an example, one of the participants stated that collaborative projects and initiatives often come to an end when initial funding runs out. Integral financing for the interprofessional team as a whole was mentioned by the participants as possible answer to this financial issue.

#### Information and communications technology (ICT)

Participants also discussed the availability of ICT-related tools to support the interprofessional collaboration. These tools could be divided into two groups: tools for communication and tools for documentation of patient information. Participants mentioned the growing supply of possibilities for health care professionals to communicate with each other from a distance via, e.g., a Skype or Adobe connection, and the rising possibilities of asynchronous communication.

*“One part of the key to successful interprofessional collaboration can be found in the implementation of ICT, and shared patient files”. (Manager, H1.8)*

Furthermore, participants introduced the use of shared information systems to document patient information and the benefit of sharing patient information. Some participants mentioned the example of the patient taking along a memory stick with personal information and the care plan to the different health care providers. In doing so, the patient is owner of his patient file and secures who will get access to the information.

## Discussion

The aim of this qualitative focus group study was to map influential factors of interprofessional collaboration (IPC) concerning patient-centred care planning in primary care, as experienced by health care professionals and experts from various backgrounds. Our findings show a variety of factors that influence the process of interprofessional care plan development. The results can be divided into 5 categories: (1) patient-related factors: active role of the patient, formulation/language of patients’ goals and wishes, member of the team; (2) professional-related factors: individual competences, domain thinking, motivation; (3) interpersonal factors related to the interaction between team members: use of discipline-specific language, knowing each other, trust, and respect; (4) organisational factors: format and composition of the team, time and efficiency, shared vision and mission, leadership, and administrative support; and (5) external factors: education, culture, law and regulations, finance, information and communications technology. Not surprisingly, most of the factors described correspond to the general theory of IPC and findings from previous published studies. This study, however, adds patient- and professional-related factors and issues directly related to interprofessional development of the patient-centred care plans. We will discuss some of these factors in more depth.

### Point of departure

Participants mentioned “knowing each other” and “motivation” as starting points and preconditions of successful care plan development. In practice, however, professionals seem to have a lack of awareness of each other’s roles, which may lead to uncertainty and breakdowns in communication [27]. Besides knowing each other, the results show the importance of building trust and respect among team members. Moreover, it becomes clear that professionals need certain competences in order to collaborate effectively. However, Frenk et al. (2010) state that professionals are falling short on appropriate competences for effective teamwork [28]. In addition, participants emphasize the importance of interprofessional education and developing competencies that will enable students to work collaboratively throughout their professional careers. There seems to be a need for health professional education programmes that are committed to integrating interprofessional education into the curricula [29, 30]

### Leadership

Our study indicates the importance of having a leader (one of the organisational factors) who prepares, structures, and organizes the interprofessional team meeting. In practice, the practice nurse or GP often fulfils the role of this chairperson. Cheater and colleagues (2005) recommend a trained, external chairperson or facilitator to structure and guide the interprofessional team meeting [31]. Their study reveals a positive effect of an external chairperson, who used strategies to encourage collaborative working, associated with improvements to care [31]. However, hiring an external leader may lead to considerable costs, and it’s unknown if the benefits of such an external leader outweigh the additional costs.

### Professional related factors

Another interesting finding is that participants experience domain thinking as a barrier to the process of shared care plan development. Baldwin (2007) sees this phenomenon of territoriality as a major challenge to IPC, in which the members of the group protect the scope and practice of their particular profession in regard to identity autonomy and accountability [32].

### Patient related factors

Our results also show the importance of involving the patients’ perspective during care plan development. Empirical evidence emerging from qualitative studies indicates that patients value an approach to care that is focused on their individual needs and facilitates their involvement in care [33]. Based on their review, D’amour et al. (2005) concluded that the patient is one of the main actors of a professional team [16]. Patient participation takes different forms, tends to vary in application, and often is not explicitly stated. There is a growing advocacy for including patients as members of the teams collaboratively managing their chronic illnesses [34]. A systematic review shows that patients with chronic diseases who participate in the decision-making process are better able to reach treatment agreement [35]. However, Safran (2003) found that for most patients in primary care, the team remains invisible. Becoming more visible as a team seems to be a challenge [36].

### Team development

Finally, our study shows that IPC related to care plan development is about teamwork and is not associated to one given moment in time but more in line with a longitudinal developing process. Depending on the level of team development, different factors have an impact on care plan development. Periodic evaluation and reflection were mentioned by participants as a method of paying attention to team development and functioning. A review of Widmer and colleagues (2009) on recent developments in reflexivity also demonstrates that reflexivity can be important to guarantee and foster team functioning [37]. Besides, periodic evaluation and reflection, they emphasize the role of leadership during team development and highlight the leader’s key role in guiding reflection and discussing processes [37].

### Strengths and limitations

The description of the results of this study should be interpreted taking into consideration the strengths and limitations of this study. One of the strengths of this study is the mixture of participants involved in the focus groups. We included health care professionals form different disciplines and experts with a variety of experiential expertise working at different facilities, which enlarged the variety of view. Furthermore, the use of an experienced and independent moderator avoided bias. In their answers, the participants seemed to stick on theory instead of personal experiences, which could be seen as a possible limitation. Moreover, the patient perspective appears to be underexposed because the patient-experts seemed to have adopted a rather professional perspective. We recognize the importance of further research on this patient perspective. Moreover, we acknowledge that we cannot ignore the possibility that conducting an extra focus group meeting could bring in additional information. However, by purposive sampling a diverse range of professionals and experts, we think this possible shortcoming has been overcome.

## Conclusions

When targeting interventions aimed at improving IPC related to care plan development in primary care, a variety of influencing factors have to be taken into account. We recommend the development of interventions or tools that are multifaceted and focus on the patient perspective as a starting point, the professional’s competences and attitudes, the interaction and communication between team members, the organisation and structuring of interprofessional team meetings, and the influence of external factors (e.g., law and regulation). The leader of the team seems to play a key role in the development and guidance of IPC. To conclude, it appears to be desirable to develop interventions with an integrated approach including these aspects. Furthermore, awareness needs to be raised for the importance of the patient perspective during care plan development. Further research on the patient perspective during care plan development seems to be desirable.

## Abbreviations

ICF, International Classification of Functioning; IPC, Interprofessional collaboration

## Additional files

Additional file 1: Background information of participants. (DOCX 16 kb) Additional file 2: Semi structured interview guide. (DOCX 14 kb)
