# Supplementary material for: Interprofessional collaboration regarding patients’ care plans in primary care: a focus group study into influential factors
Source: BMC Fam Pract. 2016 May 28;17:58. doi: 10.1186/s12875-016-0456-5 (PMC4884411; doi:10.1186/s12875-016-0456-5)
Supplement: Additional file 1: — Background information of participants. (DOCX 16 kb) [file 12875_2016_456_MOESM1_ESM.docx]

**Additional file 1:** Background information of participants

| **N** | **Identification number** | **Occupation** | **Expertise** |
| --- | --- | --- | --- |
| 1 | E1.1 | Consultant | Process management / organisational science |
| 2 | E1.2 | Researcher | Self-management |
| 3 | E1.3 | Researcher | Implementation science |
| 4 | E1.4 | Teacher general practice | Patient communication |
| 5 | E1.5 | Teacher/researcher | Interprofessional education, patient centred care |
| 6 | E1.6 | Teacher/researcher | Interprofessional teamwork, technology in care |
| 7 | E1.7 | Manager | Health care processes and logistics |
| 8 | E1.8 | Teacher/researcher | Communication and multimedia design |
| 9 | H1.1 | Practice nurse | Geriatrics |
| 10 | H1.2 | Psychologist/researcher | Neuropsychology |
| 11 | H1.3 | Manager | Patient organisation, patient perspective |
| 12 | H1.4 | Family physician | Chronic care, polypharmacy |
| 13 | H1.5 | Physical therapist | Geriatrics |
| 14 | H1.6 | Social worker | Manager social work |
| 15 | H1.7 | Occupational therapist | Practice holder, interprofessional collaboration |
| 16 | H1.8 | Manager | Manager home care, nurse |
| 17 | H1.9 | Nurse/teacher | Geriatric clinical nurse specialist |
| 18 | E2.1 | Researcher | Self-management, patient perspective |
| 19 | E2.2 | Manager | Patient organisation, informal care giving |
| 20 | E2.3 | Teacher/researcher | Communication trainer, patient professional communication |
| 21 | E2.4 | Teacher/researcher | Patient-centred care |
| 22 | E2.5 | Teacher | Patient – professional communication |
| 23 | E2.6 | Researcher | Patient communication |
| 24 | E2.7 | Researcher | Professor family practice, shared decision making |
| 25 | E2.8 | Researcher | Patient communication |
| 26 | H2.1 | Practice nurse | Geriatrics |
| 27 | H2.2 | Social worker | Youth care |
| 28 | H2.3 | Physical therapist | Geriatrics, teacher |
| 29 | H2.4 | Practice nurse | Geriatrics |
| 30 | H2.5 | Physical therapist | Psychosomatic, geriatrics, teacher |
| 31 | H2.6 | Occupational therapist | Geriatrics, psychology |
