# Supplementary material for: Interprofessional collaboration regarding patients’ care plans in primary care: a focus group study into influential factors
Source: BMC Fam Pract. 2016 May 28;17:58. doi: 10.1186/s12875-016-0456-5 (PMC4884411; doi:10.1186/s12875-016-0456-5)
Supplement: Additional file 2: — Semi structured interview guide. (DOCX 14 kb) [file 12875_2016_456_MOESM2_ESM.docx]

**Additional file 2** Semi structured interview guide

Interview guide focus groups

- Introduction
  - Introduction of the discussion leader/moderator
  - Consent for audio recording
  - Explanation of participants’ rights
  - Aim of the focus group: The aim is to explore your experience with interprofessional collaboration in the context of self-management in primary care, to explore your viewpoint about relevant factors and about facilitators and barriers.
  - Procedure, duration etc.
  - Definition self-management: Self-management is defined as “the degree to which a patient with a chronic condition is able and willing to control his or her daily life by effective management of symptoms, treatment of physical, social and psychological disease consequences, and lifestyle adjustments”.
  - Definition interprofessional collaboration: We define interprofessional collaboration as “Multiple health workers from different professional backgrounds work together with patients, families, caregivers, and communities to deliver the highest quality of care”.
  - Clarification that all information is relevant

***Main questions:***

1. *What is the first thing that comes into your mind when you think about interprofessional collaboration in primary care?*
2. *What are your experiences with interprofessional collaboration (related to the development of patient-centred care plans) in primary care?*
3. *According to you, what are relevant factors that influence the process of interprofessional collaboration (related to the development of patient-centred care plans) in primary care?*
4. *What do you experiences as a barrier/facilitator with regard to the process of interprofessional collaboration (related to the development of patient-centred care plans) in primary care?*
5. *Did you miss a relevant question/factor in this discussion?*

***Subquestions:***

- *Can you give an example?*
- *Can you explain in detail what you mean by…?*
- *Why do you experience...as a relevant factor/barrier/facilitator?*

***Summary:***

Moderator gives a summary.

- *Did I forget relevant information in this summary?*
